# Combination of 137Cs and 210Pb Radioactive Atmospheric Fallouts to Estimate Soil Erosion for the Same Time Scale

**DOI:** 10.3390/ijerph17228292

**Published:** 2020-11-10

**Authors:** Foued Gharbi, Torfa Hamad AlSheddi, Rebai Ben Ammar, Medhat Ahmed El-Naggar

**Affiliations:** 1 Department of Physics, College of Science, King Faisal University, P.O. Box 400, Al-Ahsa 31982, Saudi Arabia; talsheddi@kfu.edu.sa; 2 Unité de Radioanalyse, Centre National des Sciences et Technologies Nucléaires, Technopôle de Sidi Thabet, Sidi Thabet 2020, Tunisia; 3 Department of Biological Sciences, College of Sciences, King Faisal University, P.O. Box 400, Al-Ahsa 31982, Saudi Arabia; rbenammar@kfu.edu.sa; 4 Laboratoire des Plantes Aromatiques et Médicinales, Centre de Biotechnologie de Borj Cédria B. P 901, Hammam-lif 2050, Tunisia; 5 National Research Central Lab., GSFMO, P.O. Box 3402, Riyadh 12343, Saudi Arabia; Drnaggar@sago.gov.sa; 6 Agricultural Research Center, Plant Pathology Research Institute, Giza P.O. Box 12619, Egypt

**Keywords:** soil erosion, ^210^Pb, ^137^Cs, mass balance, cultivation, land use change

## Abstract

Naturally occurring ^210^Pb and artificial ^137^Cs fallouts are widely used as radioactive tracers for the determination of water-induced soil erosion for different time scales equal to 50 and 100 years, respectively. There exist several calibration models useful to convert the variation of the inventory of these radiotracers in cultivated soil compared to its value on non-disturbed soil to a soil erosion rate. The most comprehensive calibration models are based on a mass balance approach. In the present work, a new calibration model is proposed. It consists on the generalization of the mass balance approach to a cultivated soil subject to two successive and continuous periods of cultivation. The proposed model combines ^210^Pb and ^137^Cs fallouts for the same time scale by relaxing the constraint on ^210^Pb fallout from being used for 100 years’ time scale. The model was applied successfully to hypothetical cases and can be used to measure soil erosion rates for practical cases. It is important to note that the proposed model has two main advantages. First, the complementarity between ^210^Pb and ^137^Cs fallouts is for the same time scale and not for different time scales, as usually considered and believed in this field. Second, ^210^Pb fallout is used for time scales less than 100 years. This makes the model useful to estimate soil erosion rates for two successive periods of cultivation. To the best knowledge of the authors, the combination of ^210^Pb and ^137^Cs fallouts for the determination of soil erosion rate variation due to change in cultivation practices for the same time scale has never been developed or applied in the past.

## 1. Introduction

Rates of soil loss from agricultural land and associated soil degradation are important requirements for a successful environmental management. Rates of soil loss are strongly related to longer-term sustainability of soil resources [1]. Fallouts of ^137^Cs and^210^Pb are widely used to estimate rates of soil loss in the landscape [2,3,4,5,6,7,8]. ^137^Cs is used to produce information on erosion rates over the past 50 years [9] and ^210^Pb fallout (or ^210^Pb excess, noted as ^210^Pb_ex_) is used to provide information relating to a longer period of up to 100 years [10]. To the best knowledge of the authors, there exists only one published work estimating the change in erosion rate due to change of cultivation practices using radioactive fallouts [11]. In this work, the change of cultivation practice consisted on a shift from conventional tillage to non-tillage and the estimation of soil erosion rate change was based on ^137^Cs only and then was constrained to the time scale relative to ^137^Cs (50 years). In the present work,^210^Pb_ex_ and ^137^Cs are combined using a mass balance approach for the determination of the change in soil erosion rate due to the change in cultivation practices. Two successive and continuous periods of cultivations are assumed. To develop the model, it is necessary to relax the commonly adopted constraint on ^210^Pb_ex_ from being used for a period of 100 years. This allows for the formulation of a mass balance mathematical model using ^210^Pb_ex_ and relating its inventory at the year of measurement to the two values of erosion rates relative to the recent (after the change of cultivation practice) and the old (before the change of cultivation practice) period of cultivation. The recent period of cultivation is assumed to be comparable to 50 years. The old period can be less than 100 years. The developed mathematical model can be resolved without the need to write the corresponding complicated differential equations because each mathematical term in its solution has a clear physical meaning. This fact simplifies the use of the model in spite of the complexity of its mathematical formulation. The resolution of the model uses as input the value of the measured inventory of ^210^Pb_ex_ on the eroded location and its reference value (inventory of ^210^Pb_ex_on a non-perturbed site). If the model has a mathematical solution then the output is the erosion rate value for the recent and the old period of cultivation. Otherwise, soil deposition happened during the old and/or the recent period of cultivation. This corresponds to five different cases where ^210^Pb_ex_ has to be combined with ^137^Cs for the same time scale (50 years) to allow for the resolution of the model and to produce the soil redistribution rate (erosion or deposition rates) for each of the two periods. The proposed model was tested successfully for different hypothetical cases and can be applied on practical measurement. To the best knowledge of the authors, the procedure described in this work, which permits the estimation of the variation of the redistribution rate associated with a change in cultivation practices, represents a novel application of ^210^Pb_ex_ and ^137^Cs measurements in soil erosion investigations. This model can be re-formulated for the case of non-cultivated soils or for the case of change from cultivation to non-cultivation practice or vice versa.

## 2. Materials and Methods

The basic form of the mass balance model for an eroding location is described in detail in [9,10]. Briefly, it consists on the following differential equation:

(1) $$\frac{dA\left(t\right)}{dt}=F\left(t\right)-\left(\lambda +\frac{h}{d}\right)A\left(t\right)$$

where t is the time (y), $F\left(t\right)$ the annual deposited flux (Bq m^−2^ y^−1^), λ (y^−1^) the decay constant of the radioactive tracer (y^−1^), h the soil erosion rate (kg m^−2^ y^−1^), d the plow depth (kg m^−2^), and $A\left(t\right)$ the inventory (Bq m^−2^) of the radiotracer on the location at time t (year of the measurement). This equation assumes only one time period of cultivation, during which soil was eroded by a rate h (kg m^−2^ y^−1^) with a plow depth d (kg m^−2^). If ^137^Cs is used to determine soil erosion rate, the mass balance approach provides an estimation of the erosion rate for a window of about 50 years because the onset of ^137^Cs fallout is at 1953. If ^210^Pb_ex_ is used, it is generally constrained to provide an estimation of erosion rate for a period of about 100 years. It is, however, possible to use ^210^Pb_ex_ for shorter time periods of cultivation if needed [9,12,13]. The solution $A\left(t\right)$ of Equation (1) is:

(2) $$A\left(t\right)=A_{ref}e^{-\left(\lambda +\frac{h}{d}\right)\left(t-t_{0}\right)}+\int_{t_{0}}^{t}F\left(t\right)e^{-\left(\lambda +\frac{h}{d}\right)\left(t-t^{\prime }\right)}dt^{\prime }$$

$A_{ref}$ in this equation represents the reference inventory. In case of ^210^Pb_ex_, which is characterized by a constant flux F, the reference inventory is equal to $\frac{F}{\lambda }$ and Equation (2) becomes:

(3) $$A\left(t\right)=A_{ref}e^{-\left(\lambda +\frac{h}{d}\right)\left(t-t_{0}\right)}+(1-\frac{F}{\lambda +\frac{h}{d}}e^{-\left(\lambda +\frac{h}{d}\right)\left(t-t_{0}\right)})$$

In Equation (3), it is assumed that cultivation begins at some year $t_{0}$ (y) ($t>t_{0}$). The right-hand side of Equation (3) contains two terms. Their meaning is the following.

- $A_{ref}e^{-\left(\lambda +\frac{h}{d}\right)\left(t-t_{0}\right)}$ Inventory of ^210^Pb_ex_ contained on the location before the beginning of cultivation decreased by radioactive decay and by soil erosion during the cultivation period $\left(t-t_{0}\right)$.
- $\left(1-\frac{F}{\lambda +\frac{h}{d}}e^{-\left(\lambda +\frac{h}{d}\right)\left(t-t_{0}\right)}\right)$: Inventory of ^210^Pb_ex_ accumulated on the location during the cultivation period $\left(t-t_{0}\right)$ decreased by radioactive decay and by soil erosion.

It is important to note that each of these terms has a clear physical meaning and a specific expression. They can be written directly based on their meaning. This is very useful when dealing with multiple successive cultivation periods because it simplifies the development and the resolution of the relative complex mass balance model. In case of two successive and continuous cultivation periods, cultivation would begin from some year $t_{0}$ to some year $t_{1}$ with erosion rate $h_{1}$ and plow depth $d_{1}$ for the old cultivation period and from the year $t_{1}$ to the year t of measurement ($t_{0}<t_{1}<t$) with erosion rate $h_{2}$ and plow depth $d_{2}$ for the recent cultivation period. The inventory $A\left(t\right)$ of ^210^Pb_ex_ in this case can be expressed directly as follows.

(4) $$A\left(t\right)=A_{ref}e^{-\left(\lambda +\frac{h_{1}}{d_{1}}\right)\left(t_{1}-t_{0}\right)}e^{-\left(\lambda +\frac{h_{2}}{d_{2}}\right)\left(t-t_{1}\right)}+\frac{F}{\lambda +\frac{h_{1}}{d_{1}}}(1-e^{-\left(\lambda +\frac{h_{1}}{d_{1}}\right)\left(t_{1}-t_{0}\right)})e^{-\left(\lambda +\frac{h_{2}}{d_{2}}\right)\left(t-t_{1}\right)}\;+(1-\frac{F}{\lambda +\frac{h_{2}}{d_{2}}}e^{-\left(\lambda +\frac{h_{2}}{d_{2}}\right)\left(t-t_{1}\right)})$$

All the terms at the right-hand side of Equation (4) are comprehensive, and this is the reason their expressions can be written directly. Their meaning is explained in the following.

- $A_{ref}e^{-\left(\lambda +\frac{h_{1}}{d_{1}}\right)\left(t_{1}-t_{0}\right)}e^{-\left(\lambda +\frac{h_{2}}{d_{2}}\right)\left(t-t_{1}\right)}$: Inventory of ^210^Pb_ex_ existing on the location before the beginning of cultivation decreased by radioactive decay and by soil erosion during the old and the recent time periods of cultivation.
- $\frac{F}{\lambda +\frac{h_{1}}{d_{1}}}(1-e^{-\left(\lambda +\frac{h_{1}}{d_{1}}\right)\left(t_{1}-t_{0}\right)})e^{-\left(\lambda +\frac{h_{2}}{d_{2}}\right)\left(t-t_{1}\right)}$: Inventory of ^210^Pb_ex_ accumulated on the location during the old period of cultivation decreased by radioactive decay and by soil erosion during its accumulation along the old period and decreased during the recent period by radioactive decay and by soil erosion.
- $\left(1-\frac{F}{\lambda +\frac{h_{2}}{d_{2}}}e^{-\left(\lambda +\frac{h_{2}}{d_{2}}\right)\left(t-t_{1}\right)}\right)$: Inventory of ^210^Pb_ex_ accumulated on the location during the old time period of cultivation decreased by radioactive decay and by soil erosion during this period.

**Figure 1:**
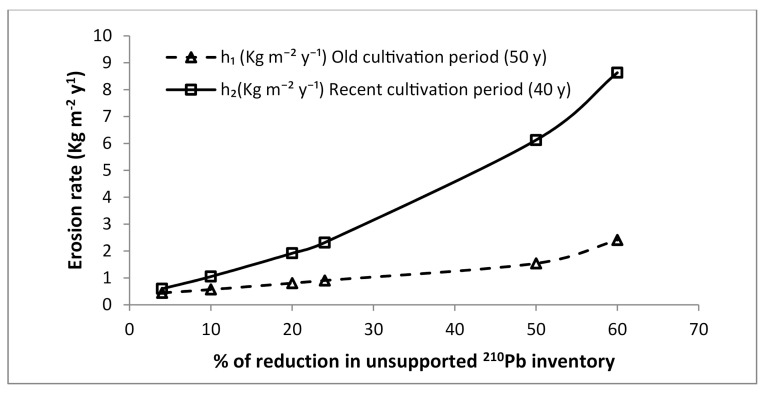
Erosion rate as a function of percentage reduction in ^210^Pb_ex_ obtained using the mass balance model (Equation (4)) for a hypothetical eroding soil subject to two successive periods of continuous cultivation.

If during the two cultivation periods, only erosion happened on the location, ^210^Pb_ex_ is sufficient as radiotracer to determine the value of erosion rate during each period using Equation (4). Figure 1 illustrates the result of a numerical test of the model (Equation (4)). The figure shows the obtained relationships between soil erosion rate and the percentage reduction in ^210^Pb_ex_ inventory relative to the local reference inventory for a hypothetical eroding soil. The old and the recent period of cultivation are assumed equal to 50 and 40 years, respectively ($\left(t-t_{1}\right)=40$ y and $\left(t_{1}-t_{0}\right)=50$ y). The following values of the employed relevant parameters were considered: $d_{1}=270$ kg m^−2^(plow depth of the old period), $d_{2}=170$ kg m^−2^ (plow depth of the recent period), and $A_{ref}=5000$ Bq m^−2^.

To resolve Equation (4), it is possible to assume that the values of erosion rates $h_{1}$ and $h_{2}$ are two unknown zeros of the function equal to the difference between the calculated inventory $A\left(t\right)$ of ^210^Pb_ex_ and the measured inventory on the location. Excel software (Microsoft, Redmond, WA, United States), through its well-known Solver numerical tool, can be used to find these zeros which determine the values of erosion rates.

The previous studied case (Will be called **case-0**) where ^210^Pb_ex_ is sufficient as a radiotracer to determine the erosion rate of each cultivation period is not unique. There exist five other cases where ^210^Pb_ex_ alone is not sufficient to determine the redistribution rates and where another radiotracer has to be used. If the recent cultivation period is in the range 40–50 years or less, ^137^Cs would be the best candidate. If the measured inventory of ^210^Pb_ex_ on the location is less than the reference inventory and Equation (4) does not have a solution, this means that the location was subject to a deposition during one of the two periods of cultivation. This corresponds to two cases:

- **Case 1:** Deposition during the old period of cultivation and erosion during the recent period with reduction of ^210^Pb_ex_ inventory.
- **Case 2:** Erosion during the old period of cultivation and deposition during the recent period with reduction of ^210^Pb_ex_ inventory.
- If the measured inventory of^210^Pb_ex_ on the location is greater than the reference inventory, there could be deposition during the two periods of cultivation or deposition during only one period and/or erosion during the other. This corresponds to three cases:
- **Case 3:** Deposition during the old period of cultivation and erosion during the recent period with excess of ^210^Pb_ex_inventory.
- **Case 4:** Erosion during the old period of cultivation and deposition during the recent period with excess of ^210^Pb_ex_inventory.
- **Case 5:** Deposition during the old period of cultivation and deposition during the recent period with excess of ^210^Pb_ex_ inventory.

One may think that ^210^Pb_ex_ can be re-used (in place of ^137^Cs) with a mass balance approach restricted to the recent period of cultivation to provide the redistribution rate of the location during the old period. This, however, is not possible because the inventory of ^210^Pb_ex_ at the beginning of cultivation of the recent period is not known, since it was altered by the cultivation during the old period. 

## 3. Results and Discussion

In the following, the combination of ^210^Pb_ex_ and ^137^Cs for the determination of the redistribution rate during the old and recent periods of cultivation is explained for each of the five described cases.

### 3.1. Case 1: Deposition during the Old Period of Cultivation and Erosion during the Recent Period with Reduction of ^210^Pb_ex_Inventory

In this case,^137^Cs provides the value of erosion rate during the recent period $\left(h_{2}\right)$ using the usual mass balance approach corresponding to one period of cultivation (the recent period). $h_{2}$ can be used to determine ^210^Pb_ex_ inventory $A\left(t_{1}\right)$ at the end of the old period of cultivation (at year $t_{1}$) by resolving the equation:

(5) $$A_{meas}\left(t\right)=A\left(t_{1}\right)e^{-\left(\lambda +\frac{h_{2}}{d_{2}}\right)\left(t-t_{1}\right)}+\frac{F}{\left(\lambda +\frac{h_{2}}{d_{2}}\right)}(1-e^{-\left(\lambda +\frac{h_{2}}{d_{2}}\right)\left(t-t_{1}\right)})$$

where $A_{meas}\left(t\right)$ is the measured inventory of ^210^Pb_ex_ on the location. It is important to note that Equation (5) is a ^210^Pb_ex_ mass balance model applied to the recent period and which uses the erosion rate $h_{2}$ produced by the use of ^137^Cs during a time period comparable to the time scale appropriate for the use of ^137^Cs. It is in that sense that the combination of ^210^Pb_ex_ and ^137^Cs for the same time scale is understood in the present approach. All the combination sof ^210^Pb_ex_ and ^137^Cs relative to each of the five different cases described previously are for the same time scale in that sense. As a numerical test of the model for this case, Table 1 shows the obtained erosion rate values for the recent period from the resolution of Equation (5) for three different hypothetical values of $A_{meas}\left(t\right)$ and $A\left(t_{1}\right)$. It is clear from the three examples illustrated in Table 1 that erosion rate during the recent period was sufficiently high to reduce the inventory.

The calculation procedure of the deposition rate during the old period is well known [9,10] and is described briefly here. It is based on the calculation of the concentration of ^210^Pb_ex_ in the mobilized sediment $C_{e}\left(t^{\prime }\right)$ (Bq kg^−1^), which depends on the erosion of a proportion of the annually deposited ^210^Pb fallout from the eroding soil profile (from the up slope in general) prior to its incorporation by tillage, and on the removal of the accumulated fallout ^210^Pb that is stored in the plow layer. The knowledge of $C_{e}\left(t^{\prime }\right)$ allows for the estimation of ^210^Pb_ex_ concentration of deposited sediment $C_{d}\left(t^{\prime }\right)$ at year *t’*(y) on the location. The relation between $C_{d}\left(t^{\prime }\right)$ and $C_{e}\left(t^{\prime }\right)$ depends on the erosion rate $h_{1}$ and on the particle-size correction factor that reflects differences in grain-size compositions of mobilized and deposited sediment and is defined as the ratio of ^210^Pb_ex_ concentration of deposited sediment to that of the mobilized sediment. The correction factor $P^{\prime }$ is generally less than 1 [9].

(6) $$A_{ex}\left(t_{1}\right)=\int_{t_{0}}^{t_{1}}R_{1}^{\prime }C_{d}\left({t^{\prime }}^{}\right)e^{-\lambda \left(t_{1}-t^{\prime }\right)}dt^{\prime }$$

The excess inventory $A_{ex}\left(t_{1}\right)$ of ^210^Pb_ex_ at the end of the old period of cultivation is equal to the subtraction of the reference inventory from the inventory $A\left(t_{1}\right)$ determined from Equation (5). It is used to determine the deposition rate $R_{1}^{\prime }$ (kg m^−2^ y^−1^) according to Equation (6) [9,10].

### 3.2. Case 2: Erosion during the Old Period of Cultivation and Deposition during the Recent Period with Reduction of^210^Pb_ex_Inventory

In this case, the combination of ^137^Cs and ^210^Pb_ex_ is as follows. The deposition rate $R_{2}^{\prime }$ is determined using ^137^Cs based on the knowledge of the erosion rate at the up slope of the transect (determined using ^137^Cs or ^210^Pb_ex_, or both, depending on the situation). The ^210^Pb_ex_ inventory accumulated during the recent period of cultivation $A_{ex}^{recent}$ is determined using the equation [9,10]:

(7) $$A_{ex}^{recent}\left(t\right)=\int_{t_{1}}^{t}R_{2}^{\prime }C_{d}({t^{\prime }}^{})e^{-\lambda \left(t_{1}-t^{\prime }\right)}dt^{\prime }$$

where $C_{d}\left(t^{\prime }\right)$ is the ^210^Pb_ex_ concentration of deposited sediment at time *t’*(y) on the location determined using the concentration of ^210^Pb_ex_ on the mobilized sediment due to erosion at the up slope of the transect. The subtraction of $A_{ex}^{recent}$ from the measured inventory of^210^Pb_ex_ at the time of measurement increased by decay correction with the time length of the recent period (multiplied by $e^{\lambda \left(t-t_{1}\right)}$) provides the inventory $A^{old}\left(t_{1}\right)$ of ^210^Pb_ex_ at the end of the old period. The inventory at the end of the old period should be less than the reference inventory because the soil was eroded during the recent period in the present case. Erosion rate $h_{1}$ during the old period is obtained by resolving the following mass balance equation restricted to the recent period.

(8) $$A^{old}\left(t_{1}\right)=A_{ref}e^{-\left(\lambda +\frac{h_{1}}{d_{1}}\right)\left(t_{1}-t_{0}\right)}+\frac{F}{\lambda +\frac{h_{1}}{d_{1}}}(1-e^{-\left(\lambda +\frac{h_{1}}{d_{1}}\right)\left(t_{1}-t_{0}\right)})$$

The first term on the right-hand side of Equation (8) represents the reference inventory of^210^Pb_ex_ decreased by decay and by erosion during the old period, and the second term represents the accumulated ^210^Pb_ex_ during the old period decreased by decay and by erosion. It is clear in this case that ^137^Cs and ^210^Pb_ex_ are used for the same time scale. Table 2 shows hypothetical values of $A^{old}\left(t_{1}\right)$ and obtained erosion rates relative to the old period (satisfying Equation (8)).

### 3.3. Case 3: Deposition during the Old Period of Cultivation and Erosion during the Recent Period with Excess of ^210^Pb_ex_ Inventory

In this case, a reduction of ^137^Cs inventory should be observed since soil was eroded during the recent period. ^137^Cs is then used to determine the erosion rate $h_{2}$ during the recent period. The inventory of ^210^Pb_ex_ at the end of the old period $A_{}^{old}\left(t_{1}\right)$ can be calculated by solving the equation:

(9) $$A_{meas}\left(t\right)=A_{}^{old}\left(t_{1}\right)e^{-\left(\lambda +\frac{h_{2}}{d_{2}}\right)\left(t-t_{1}\right)}+\frac{F}{\lambda +\frac{h_{2}}{d_{2}}}(1-e^{-\left(\lambda +\frac{h_{2}}{d_{2}}\right)\left(t_{1}-t_{0}\right)})$$

where $A_{meas}\left(t\right)$ is the measured ^210^Pb_ex_ inventory at the end of the recent period. $A_{}^{old}\left(t_{1}\right)$ is expected to be greater than the reference inventory in the present case. The deposition rate $R_{1}^{\prime }$ during the old period is determined based on the knowledge of the erosion rate $h_{1}$ at the up slope and after calculating the concentration of ^210^Pb_ex_ of the mobilized sediment as done in case 2 for the recent period.

### 3.4. Case 4: Erosion during the Old Period of Cultivation and Deposition during the Recent Period with Excess of ^210^Pb_ex_Inventory

In this case,^137^Cs is used to determine deposition rate $R_{2}^{\prime }$ on the location during the recent period.^210^Pb_ex_ inventory accumulated on the location during the recent period of cultivation $A_{ex}^{recent}$ is determined using Equation (7). The subtraction of $A_{ex}^{recent}$ from the measured inventory of ^210^Pb_ex_ at the year of measurement, increased by decay correction with the time length of the recent period (multiplied by $e^{\lambda \left(t-t_{1}\right)}$), provides the inventory of ^210^Pb_ex_ at the end of the old period $A_{}^{old}\left(t_{1}\right)$. The determination of erosion rate during the old period is obtained using Equation (8).

### 3.5. Case 5: Deposition during the Old Period of Cultivation and Deposition during the Recent Period with Excess of ^210^Pb_ex_Inventory

In this case,^137^Cs is used to determine ^210^Pb_ex_ inventory at the end of the old period $A_{}^{old}\left(t_{1}\right)$, as described in case 2. $A_{}^{old}\left(t_{1}\right)$ is then subtracted to the reference inventory of ^210^Pb_ex_ to estimate the ^210^Pb_ex_ inventory $A\left(t_{1}\right)$ on the location at time $t_{1}$. $A\left(t_{1}\right)$ is expected to be greater than the reference inventory, indicating that the situation is corresponding to case 5.

(10) $$A_{ex}^{recent}\left(t\right)=\int_{t_{0}}^{t_{1}}R_{1}^{\prime }C_{d}({t^{\prime }}^{})e^{-\lambda \left(t_{1}-t^{\prime }\right)}dt^{\prime }$$

Using the erosion rate at the up slope of the transect during the recent period and ^210^Pb_ex_ concentration of deposited sediment $C_{d}\left(t^{\prime }\right)$ at time *t’*, the deposition rate $R_{1}^{\prime }$ during the old period can be determined on the location through Equation (10).

## 4. Conclusions

A new mass balance approach model useful for the determination of soil redistribution rates relative to two successive and continuous periods of soil cultivation is proposed. The model is based on the combination of ^210^Pb_ex_and^137^Cs for the same time scale where excess or reduction of ^210^Pb_ex_inventory at the year of measurement is known. The model can be applied on any soil subject to two successive and continuous time periods of cultivation where the recent period is comparable to the time scale appropriate to^137^Cs. ^210^Pb_ex_ is used without being constrained to 100 years, as commonly assumed, which allows for the use of the two radiotracers for the same time scale. Successful tests of the model for hypothetical cases were performed. The model can be applied in practical cases and can provide important information on soil redistribution rates needed to study the environmental impact of the change of soil cultivation procedures. The proposition of a model combining ^210^Pb_ex_ and ^137^Cs for the same time scale and predicting the variation of the redistribution rate of cultivated soils due to change of cultivation procedure in the present work constitutes a new method of estimation of soil redistributing rates with ^210^Pb_ex_ and ^137^Cs.

## Figures and Tables

**Table 1:** Examples of possible values of the inventories $A_{meas}\left(t\right)$, $A\left(t_{1}\right)$, and the erosion rates $h_{2}$ compatible with Equation (2), assuming $d_{2}$ is equal to 170 kg m^−2^.

$A_{meas}\left(t\right)$ (Bq m−2)	$A\left(t_{1}\right)$ (Bq m−2)	$h_{2}$ (kg m^−2^ y^−1^)
4739	6000	0.8
4385	5500	1.2
4180	7000	2.1

**Table 2:** Obtained erosion rate values for the old period according to case 2 assuming $d_{1}$ is equal to 270 kg m^−2^.

$A^{old}\left(t_{1}\right)$ (Bq m−2)	$h_{1}$ (kg m^−2^ y^−1^)
4907	0.2
4773	0.5
4480	1.2
